# Lansoprazole attenuates cyclophosphamide-induced cardiopulmonary injury by modulating redox-sensitive pathways and inflammation

**DOI:** 10.1007/s11010-023-04662-x

**Published:** 2023-01-31

**Authors:** Emad H. M. Hassanein, Esam O. Kamel, Wail M. Gad-Elrab, Mohammed A. Ahmed, Zuhair M. Mohammedsaleh, Fares E. M. Ali

**Affiliations:** 1 Department of Pharmacology and Toxicology, Faculty of Pharmacy, Al-Azhar University, Assiut Branch, Assiut, 71524 Egypt; 2 Department of Medical Histology and Cell Biology, Faculty of Medicine, Al-Azhar University, Assiut, Egypt; 3 Department of Human Anatomy & Embryology, Faculty of Medicine, Al-Azhar University, Assiut, Egypt; 4 Pathology Department, Faculty of Medicine, Al-Azhar University, Assiut, Egypt; 5 Department of Medical Laboratory Technology, Faculty of Applied Medical Sciences, University of Tabuk, Tabuk, 71491 Kingdom of Saudi Arabia

**Keywords:** Lansoprazole, Cyclophosphamide, Cardiopulmonary, Nrf2/HO-1, PI3K/AKT, NF-κB

## Abstract

Cyclophosphamide (CPA) is a classical chemotherapeutic drug widely used as an anticancer and immunosuppressive agent. However, it is frequently associated with significant toxicities to the normal cells of different organs, including the lung and heart. Lansoprazole (LPZ), a proton pump inhibitor (PPI), possesses antioxidant and anti-inflammatory properties. The current study investigated how LPZ protects against CPA-induced cardiac and pulmonary damage, focusing on PPARγ, Nrf2, HO-1, cytoglobin, PI3K/AKT, and NF-κB signaling. Animals were randomly assigned into four groups: normal control group (received vehicle), LPZ only group (Rats received LPZ at a dose of 50 mg/kg/day P.O. for 10 days), CPA group (CPA was administered (200 mg/kg) as a single i.p. injection on the 7th day), and cotreatment group (LPZ plus CPA). Histopathological and biochemical analyses were conducted. Our results revealed that LPZ treatment revoked CPA-induced heart and lung histopathological alterations. Also, LPZ potently mitigated CPA-induced cardiac and pulmonary oxidative stress through the activation of PPARγ, Nrf2/HO-1, cytoglobin, and PI3K/AKT signaling pathways. Also, LPZ effectively suppressed inflammatory response as evidenced by down-regulating the inflammatory strategic controller NF-κB, MPO, and pro-inflammatory cytokines. The present findings could provide a mechanistic basis for understanding LPZ's role in CPA-induced cardiopulmonary injury through the alleviation of oxidative stress and inflammatory burden.

## Introduction

Chemotherapeutic drugs are widely used to treat a variety of cancers. However, their clinical use is usually associated with multiple organ toxicities. Systematic chemotherapy’s lung and heart intoxications are major health problems [1–4]. Cyclophosphamide (CPA) is an example of a classical chemotherapeutic drug and is widely used as an anticancer and immunosuppressive agent. However, it is frequently associated with significant toxicities of the normal cells of different organs of the body systems [5, 6]. CPA metabolites (hydroxy cyclophosphamide and acrolein) induce cardiotoxicity by increasing ROS and decreasing the antioxidant defense mechanisms [7, 8]. Similarly, the lung metabolites cause significant lung intoxication [9].

The nuclear factor erythroid 2-related factor 2 (Nrf2) transcription factor is a crucial factor in redox regulation in cardiovascular diseases [10] as well as lung diseases [11]. It functions as a target nuclear receptor against oxidative stress. Nrf2 regulates a wide range of target genes through transcriptional regulation. After exposure of cells to ROS and electrophiles, the Nrf2/heme oxygenase-1 (HO-1) system regulates many detoxifications and antioxidant enzymes that potently protect against pulmonary [12] and heart [13] injuries. Therefore, the elimination of ROS might be the molecular basis of Nrf2-mediated anti-inflammation and anti-oxidation [14, 15]. Also, the nuclear receptor peroxisome proliferator-activated receptor gamma (PPARγ) is essential in treating cardiovascular and lung diseases. Endogenous and exogenous ligands-agonists stimulate PPARγ, which is involved in the activation of several cellular signaling pathways involved in the oxidative stress response, such as the phosphatidylinositol 3′-kinase/protein kinase B (PI3K/AKT) signaling pathway [16–18]. Several studies reported that the activation of PI3K/AKT plays a key role in protection against heart and lung oxidative stress and inflammation depending on its involvement in different models of heart injury, such as diabetic cardiomyopathy [19] and doxorubicin-induced cardiomyopathy [20] as well as lung injuries, such as lung ischemia–reperfusion injury [21], sepsis-induced pulmonary injury [22], and others.

Another interesting point is the activation of nuclear factor-*kappa* B (NF-κB) due to ROS overproduction, resulting in myocardial injury [23]. The NF-κB protein complex is a redox-sensitive protein complex that plays a crucial role in inflammation. Activated NF-κB causes myocardial inflammation by promoting the transcription and release of inflammatory mediators, such as tumor necrosis factor-alpha (TNF-α) [23, 24].

Lansoprazole (LPZ) is a proton pump inhibitor (PPI) that inhibits gastric acid secretion by binding to the proton pump in gastric parietal cells in an irreversible manner [25]. Several studies demonstrated the dual antioxidant and anti-inflammatory effects of LPZ [26–28]. Based on the explanation mentioned above, our study aimed to investigate the cardiopulmonary protective, antioxidant and anti-inflammatory effects of LPZ and to explore the underlined molecular mechanisms by studying the involvement of PPARγ, Nrf2/HO-1, cytoglobin, PI3K/AKT, and NF-κB signals in these protective effects.

## Material and methods

### Drugs and chemicals

Lansoprazole (LPZ) and CPA were purchased from Sigma-Aldrich (USA). Serum glutamic oxaloacetic transaminase (SGOT), alkaline phosphatase (ALP), lactate dehydrogenase (LDH), and creatine kinase-MB (CK-MB) kits were purchased from SPAINREACT Company (Spain). Rat TNF-α ELISA kit was purchased from ELABSCIENCE Company (USA). PPARγ, Nrf2, HO-1, cytoglobin, PI3K, AKT, p-AKT, p-p65, vascular cell adhesion molecule-1 (VCAM-1), and β-actin antibodies were purchased from Santa Cruz (USA).

### Animals

Thirty-two adult male Wistar rats (180–200 g) were obtained from the animal house unit of Assiut University, Egypt. Rats were kept at a constant temperature of 24 ± 2 °C and a 12-h light/12-h dark cycle, with free access to a standard diet and water. All animal-related experimental procedures were approved by the Faculty of Pharmacy Ethics Committee of Al-Azhar University, Egypt (Approval number: ZA-AS/PH/30/C/2022), following the National Institutes of Health Guide for the Care and Use of Laboratory Animals.

### Experimental design

The animals were allocated randomly into four groups, each with eight animals.

- **Group I (Normal control):** Rats received the vehicle (0.5% CMC) p.o. for 10 days.
- **Group II (LPZ):** Rats received the LPZ at a dose of 50 mg/kg/day [29] suspended in 0.5% CMC) p.o. for 10 days.
- **Group III (CPA):** Rats received a single i.p. injection of CPA at a dose of 200 mg/kg [30, 31] on the 7th day.
- **Group IV (LPZ + CPA):** Rats received LPZ (50 mg/kg p.o.) for 10 days and were injected with CPA (200 mg/kg i.p.) on the 7th day.

Under ketamine anesthesia (100 mg/kg i.p.) [32], blood, heart, and lung tissue samples were collected after 24 h after the last dose to determine various histological, biochemical, and molecular markers. Centrifugation at 1000 g for 10 min was used to prepare the serum. Immediately after sacrifice, the lung and heart were excised and divided into several sections. One section was used for histopathological and immunohistochemical examinations by soaking in 10% neutral-buffered formalin. Another section was frozen quickly in liquid nitrogen and then stored in lysis buffer until they were processed for western blot analysis. The last part of the heart and lung samples was used to prepare tissues homogenate (20%) and assess oxidative stress and inflammation biomarkers in pulmonary and cardiac homogenates.

### Histopathological examinations

The method for histopathological evaluation described by Bancroft and Gamble (2008) was used [33]. Biopsies from the heart and lungs were embedded in paraffin after being fixed in a 10% neutral-buffered formalin solution. Cleaning, dehydrating with alcohol, clearing in xylene, and embedding in paraffin were all done on the samples. For histopathological evaluation, 4-µm-thick sections were cut and stained with hematoxylin and eosin (H&E) or Periodic acid–Schiff (PAS). Sections of lungs from each group were examined by two examiners. The histopathological changes in lung tissues were assessed semiquantitatively depending on the following items: (1) alveolar edema; (2) inter-alveolar edema; (3) thickness of the alveolar septum; (4) inflammatory cells infiltration; (5) extravasation of RBCs; and (6) emphysematous changes. The scoring was designed as follows: − no changes, + mild alterations, + + moderate alterations, and + + + severe alterations, and the results are tabulated (Table 1).

**Table 1:** Grading of histopathological changes in 10 fields of lung sections

Findings	Control	LPZ	CPA	CPA + LPZ
Alveolar edema	–	–	+ +	–
Inter-alveolar edema	–	–	+ +	+
Alveolar septum thickness	–	–	+ + +	+ +
Inflammatory infiltrate	–	–	+ + +	+
Extravasation of RBCs	–	+	+ +	+
Emphysematous changes	–	–	+ + +	+

(–), no deviation from the normal structure, (+) mild injurious effect, (+ +); moderate, and (+ + +); severe

After sacrificing the rats, sections of hearts from each group were examined by two examiners. Scoring of histomorphology was done by following the scoring system used by Patil, Singh, Mahajan, Patil, Ojha, and Goyal [29], and accordingly, we graded the histomorphology as (-); normal structure, (+) mild injurious effect, (+ +); moderate and (+ + +); severe as shown in Table 3.

### Biochemical investigations

#### Determination of serum cardiac function biomarkers

Serum SGOT, ALP, LDH, and CK-MB levels were measured using commercially available kits. All the assays were carried out in accordance with the manufacturer’s guidelines.

#### Investigation of oxidative stress biomarkers

The intensity of the yellow color produced by the reaction with Ellman’s reagent was used to determine the amount of reduced glutathione (GSH) in the lung and heart tissues [34], while lipid peroxidation was measured as thiobarbituric acid reacting substances and expressed as malondialdehyde (MDA) content [35]. Besides, the cardiac and pulmonary enzymatic activities of superoxide dismutase (SOD) were assessed at 420 nm using the methods described by Marklund [36].

#### Assessment of TNF-α and MPO levels

The levels of TNF-α in the heart and lung were measured using ELISA kits, according to the manufacturer’s instructions and based on the previously described principles [37]. In addition, the cardiac and pulmonary enzymatic activity of myeloperoxidase (MPO), a neutrophil infiltration biomarker, were assessed using Manktelow and Meyer [38] method.

#### Western blotting

RIPA lysis buffer was used to extract protein from lung and heart tissues, along with a protease inhibitor cocktail. For the separation of equivalent volumes of extracted proteins (50 µg of total protein) using the Bradford method [39] from all studied groups. After electrophoresis, the SDS gel was transferred to a PVDF membrane using semi-dry transfer methods [40]. Then, membranes were blocked for 1 h with 5% nonfat dry milk, tris-buffered saline, and 0.1% Tween 20 and then incubated overnight at 4 °C with the primary monoclonal anti-mouse antibodies anti-PI3K, anti-AKT, anti-p-AKT, anti-p-p65, anti-VCAM-1, and anti-β-actin. After that, the membranes were incubated for 1 h at room temperature with the appropriate secondary antibodies. The amounts of PI3K, AKT, p-AKT, p-p65, VCAM-1, and β-actin were estimated in the pulmonary and cardiac samples using the image analysis software (ImageJ software).

#### Immunohistochemical examination

The endogenous peroxidase activity was inhibited for 10 min using 3% H_2_O_2_. The sections were then antigen retrieved by incubating them for 30 min at 121 °C in 10-mM citrate buffer, followed by blocking them in 5% bovine serum albumin in tris-buffered saline. Antibodies against PPARγ, Nrf2, HO-1, and cytoglobin were used to probe the tissue sections overnight at 4 °C. After that, the secondary antibody was incubated for 15 min. The bound antibody complex was identified by the diaminobenzidine (DAB) substrate reaction and counterstaining with hematoxylin. The labeling was examined and analyzed using ImageJ to determine the mean area percentage of PPARγ, Nrf2, HO-1, and cytoglobin immune-positive reactions [32].

### Statistical analysis

The mean and standard error of the mean are used to present the findings (SEM). A one-way analysis of variance (ANOVA) was used to detect statistical significance between groups, followed by Tukey’s post hoc test. At a probability of less than 0.05, statistical significance between values was considered. GraphPad Software Inc., USA, version 7, was used to conduct the statistical analysis.

## Results

### Effect of LPZ on pulmonary and cardiac injury induced by CPA

#### Effect of LPZ on pulmonary injury induced by CPA

**Fig. 1:**
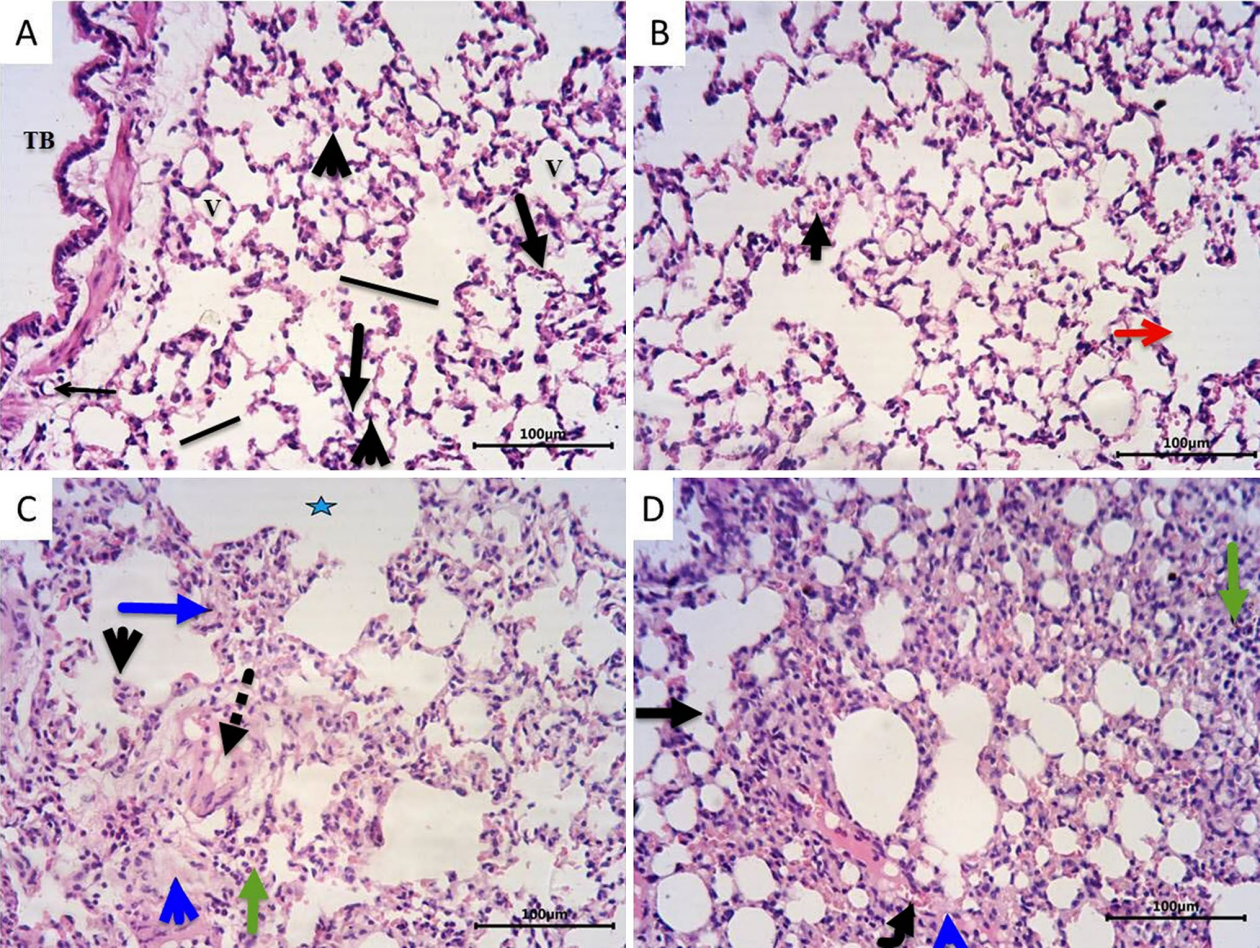
Photomicrographs of sections from rat lungs stained with H and E. **A** Representative section from the control group showed normal lung structure with normal average-sized patent alveoli (V), alveolar ducts (straight line), and normal terminal bronchioles (TB). The alveoli have thin walls and are lined by flat type 1 pneumocytes (thick arrow) and cubical type 2 pneumocytes (black arrowhead). Normal small non-engorged blood capillaries (thin arrow) are also present. **B** Section from LPZ-treated group showing normal lung morphology as control group except for slight widening of a few alveoli (red arrow) and few extravasated RBCs (short arrow).** C** Section from rat lungs treated with CPA showing alveolar obstruction by edema fluid (dotted arrow), swelling of type 2 pneumocytes (black arrowhead), thickening of the alveolar septa (blue arrow), inter-alveolar edema (blue arrowhead), acute and chronic inflammatory cell infiltrate (green arrow), and dilated alveoli with emphysematous changes (blue star). Treating CPA rats with LPZ (D) showed restricted damage of a few alveolar walls with a widening of some alveoli (transverse black arrow), minimal edema (blue arrowhead), few inflammatory cells infiltrate (green arrow), and congested capillaries (curved arrow). Scale bar = 100 µm

**Fig. 2:**
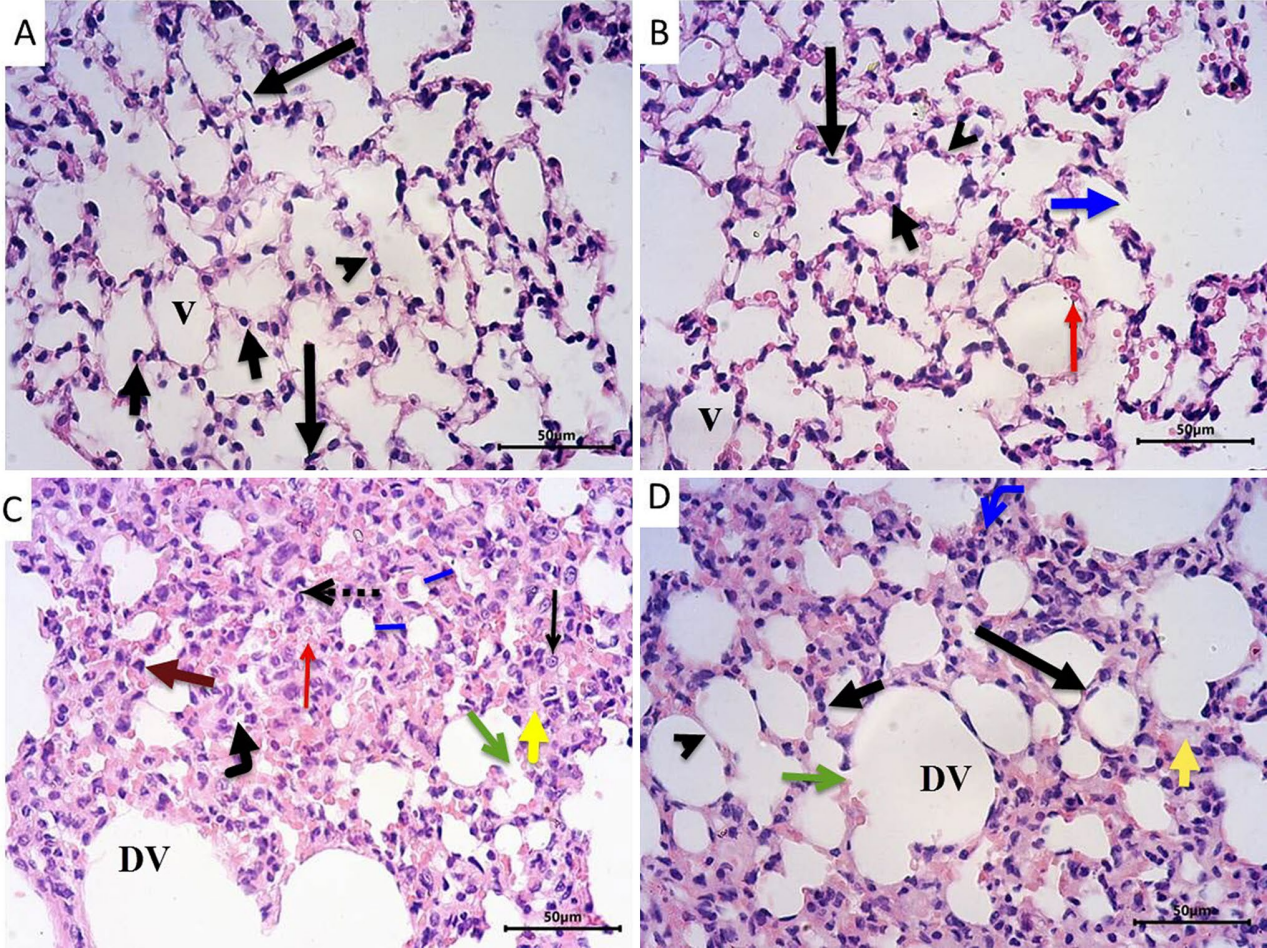
Photomicrographs of sections from lungs stained with H and E. **A** Representative section from control group showing the normal sponge-like structure of lung alveoli. Most alveoli (V) are patent and separated by thin alveolar septa (arrowhead). The alveoli are lined by flat type 1 pneumocytes (long arrow) with flat nuclei and cubical type 2 pneumocytes (short arrow) with rounded nuclei. **B** LPZ-treated rats showed normal alveolar morphology as in the control group where the alveoli (V) are lined with flat type 1 pneumocytes (short arrow) and cubical type 2 pneumocytes (long arrow) with thin separating alveolar septa (arrowhead). But some alveoli are widened with the destruction of the alveolar wall (blue arrow) and a few extravasated RBCs (red arrow). **C** Section from the CPA group showing dense inflammatory infiltrates in the form of polymorphonuclear leukocytes (dotted arrow), histiocytes (thin arrow), and extravasated RBCs (red arrow). Some alveoli are dilated (DV) and others are collapsed (curved arrow). In many areas, the alveolar septa are thick (blue straight line) with inter-alveolar edema (yellow arrow), and in other areas, the septa are destructed (green arrow). Type II pneumocytes are swollen in many areas (brown arrow). **D** Sections from rats treated with CPA and LPZ show noticeable improvement of alveolar structure where the alveoli are lined with flat type I pneumocytes (long arrow) with flat nuclei and cubical type II pneumocytes (short arrow) with rounded nuclei, but few alveoli look wide (DV) with a destructed alveolar wall (green arrow), inter-alveolar edema (yellow arrow), and mixed inflammatory cell infiltrate (curved blue arrow). Scale bar = 50 µm

Histopathological examination of sections from the lungs of the control group (Figs. 1A, 2A) revealed normal structural morphology of the lungs. The alveoli showed a sponge-like appearance with thin alveolar septa. Alveoli and alveolar ducts were patent with normal sizes and were lined by flat type 1 pneumocytes with flat nuclei and cubical type 2 pneumocytes with rounded nuclei with few alveolar macrophages. Normally looked patent-terminal bronchioles and small non-engorged blood capillaries were also present. Sections from LPZ-treated rats (Figs. 1B, 2B) showed normal lung morphology as documented in the control group, except for the slight widening of a few alveoli and extravasated RBCs. On the other hand, sections from CPA-treated rats (Figs. 1C, 2C) showed severe morphological abnormalities in the form of prominent congestion, edema, and extravasation of RBCs, together with dense inflammatory infiltrate. These inflammatory infiltrates mixed and formed macrophages, lymphocytes, plasma cells, and neutrophils. Alveoli were narrowed and compressed with thickening and expansion of the alveolar septa in many areas, and in other areas, the alveolar septa were destructed. Foci of dilated alveoli and compensatory emphysema were also noticed. Swelling of type 2 pneumocytes was observed in many areas. Interestingly, treating CPA rats with LPZ (Figs. 1D, 2D) showed noticeable improvement in the alveolar morphology with the slight remodeling of the alveolar walls and empty alveolar lumens. The alveoli were patent and separated by thin alveoli. They were lined with flat type I and cubical type II pneumocytes. However, still, there is mild damage to the alveolar walls and some dilated alveoli. Also, moderate congestion, edema, extravasation of RBCs, and mixed inflammatory infiltrate were still present. The severity of histopathological observations in lung tissues from all groups was tabulated (Table 1) and reported an improvement in CPA + LPZ-treated rats compared to CPA-only treated rats.

**Fig. 3:**
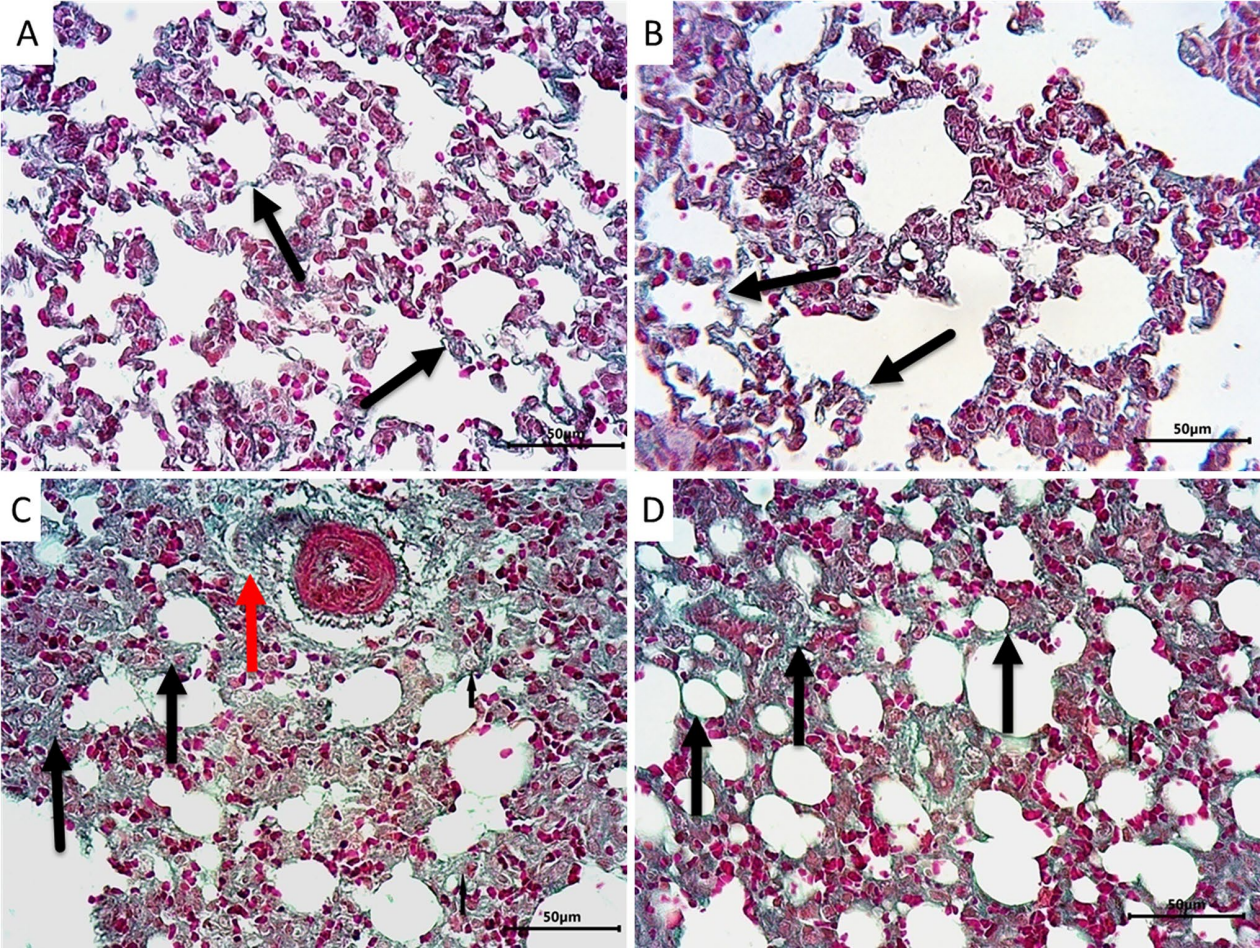
Photomicrographs of rat lungs stained with Masson’s Trichrome demonstrating collagen fibers deposition presented by green color. Sections from control (**A**) and LPZ-treated (**B**) animals show normal alveolar structure and minimal collagen fibers deposition around the alveoli (black arrows). Lung section from CPA-treated rats (**C**) shows massive collagen deposition in the thickened alveolar septa (black arrows) and around blood vessels (red arrows). In rats treated with CPA and LPZ (D), the deposition of collagen fibers around the alveoli apparently decreased (black arrows). Scale bar = 50 µm

The lung sections were stained with Masson’s Trichrome to evaluate the amount of collagen fibers deposition in the lung interstitium. Sections from control (Fig. 3A) and LPZ-treated (Fig. 3B) groups showed normal alveolar structure and minimal collagen fibers deposition around the alveoli. Administration of CPA (Fig. 3C) caused massive deposition of collagen fibers in the thickened alveolar septa and around blood vessels. On the other hand, treating CPA rats with LPZ (Fig. 3D) decreased the amount of collagen fibers deposition in the alveolar septa.

#### Effect of LPZ on cardiac injury induced by CPA

##### Effect of LPZ on serum cardiac function biomarkers

Serum levels of SGOT, ALP, LDH, and CK-MB were assessed as indicative biomarkers for heart function to evaluate the impact of LPZ against CPA-induced cardiac injury. Compared to normal control rats, CPA injection resulted in a significant increase in serum SGOT, ALP, LDH, and CK-MB levels. In contrast, oral administration of LPZ potently lowered the SGOT, ALP, LDH, and CK-MB levels compared to untreated rats, as shown in Table 2.

**Table 2:** Effect of lansoprazole on serum SOGT, LDH, ALP, and CK-MB levels after CPA-induced cardiopulmonary syndrome

		Normal	LPZ	CPA	LPZ + Cd
Serum markers	SGOT (U/L)	108.8 ± 4.89	97.5 ± 3.91	315.7 ± 19.60^*^	194 ± 6.15^*,**^
	LDH (U/L)	551 ± 70.48	617.1 ± 52.99	2730 ± 120.50^*^	1571 ± 105.70^*,**^
	ALP (U/L)	253.1 ± 17.21	251.3 ± 22.37	1370 ± 98.39^*^	357.5 ± 27.63^**^
	CK-MB (U/L)	114.4 ± 22.34	96.42 ± 4.83	1146 ± 83.73^*^	537.5 ± 71.24^*,**^

Results are presented as mean ± S.E.M (*n* = 8). Data analysis was performed statically using one-way ANOVA. ^*^P < 0.05 Vs. normal control group. ^**^P < 0.05 Vs. CPA control group

##### Effect of LPZ on cardiac histopathological abrasions induced by CPA

**Fig. 4:**
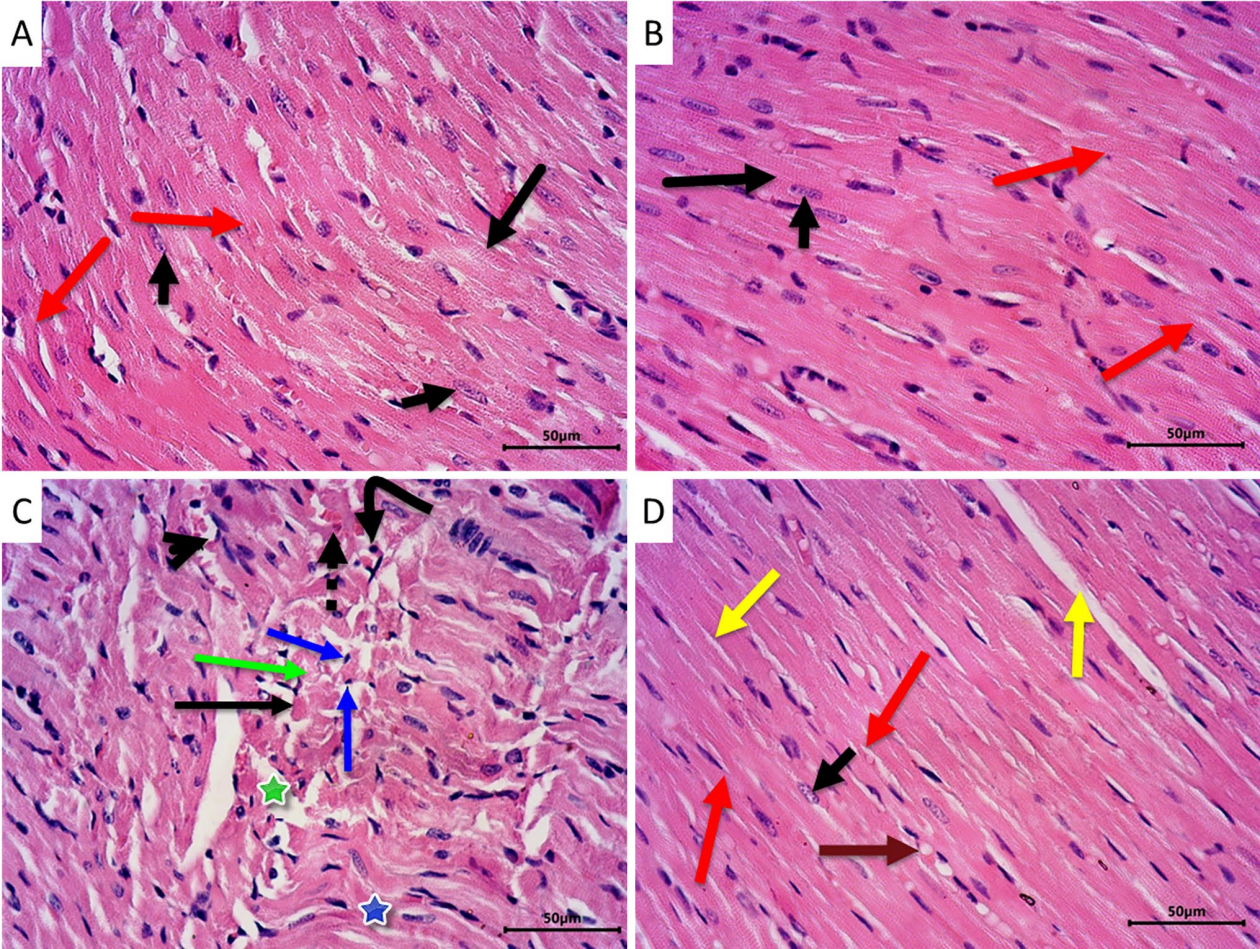
Photomicrographs of sections from rat hearts stained by H&E. Representative sections from control (**A**) and LPZ (**B**) groups showing normal histoarchitecture of the myocardium. The cardiac muscle is formed of long branching and anastomosing (red arrow) myocytes having vesicular centrally located nuclei (short arrow) and acidophilic cytoplasm and exhibiting apparent cross-striations (long arrow). Section from CPA-treated rats **C** showing dilated congested blood capillaries (arrowhead), extravasated RBCs (dotted arrow), and scattered inflammatory cells (curved arrow). The cardiac muscle fibers are distorted and show areas of waviness and splitting (blue star). Many muscle fibers have pyknotic nuclei (blue arrow) and disintegrated cytoplasm (green arrow). Foci of coagulative necrosis (thin black arrow) and loss of myocardial cellular constituents (green star) could be observed. On the other hand, treating the CPA rats with LPZ **D** showed apparent improvement in myocardial structure. Most muscle fibers are branching and anastomosing (red arrow) and have intact acidophilic cytoplasm and vesicular centrally located nuclei (short arrow). Some fibers show mild focal fatty degeneration (brown arrow) and myocardial splitting (yellow arrow). Scale bar = 50 µm

Histopathological examination of sections from rat hearts of control (Fig. 4A) and LPZ (Fig. 4B) revealed normal heart morphology. The myocardium showed a normal appearance, with branched fibers exhibiting cross-striations. The fibers had acidophilic cytoplasm and centrally located vesicular nuclei. The blood capillaries between the fibers were hardly visible without any congestion or extravasation of RBCs. On the other hand, sections from the heart of CPA-treated rats (Fig. 4C) showed severe morphological abnormalities in the form of congestion, edema, and extravasation of RBCs together with inflammatory infiltrate. Myocardial fibers were distorted, wavy, and splitted in many areas. Severe degenerative changes in the form of vacuolization and fatty changes were present. Early nuclear and cytoplasmic necrotic changes were also evident. Areas of coagulative necrosis and disintegration of cardiac myocytes were also seen in the heart sections of this group. Interestingly CPA rats treated with LPZ (Fig. 4D) showed valuable improvement in the morphological abnormalities with a significant reduction in degenerative changes. Most muscle fibers were branched and had intact acidophilic cytoplasm and vesicular centrally located nuclei. Only mild focal fatty changes and a few areas of myocardial splitting were observed. The severity of histopathological observations in heart tissues from the four groups was tabulated (Table 3) and revealed an improvement in CPA + LPZ-treated rats compared to CPA-only treated rats.

**Table 3:** The severity of histopathological changes in heart sections

Findings	Control	LPZ	CPA	CPA + LPZ
Congested vessels	–	–	+ + +	+
Extravasated RBCs	–	–	+ + +	+
Edema	–	–	+ + +	+
Inflammation	–	–	+ +	+
Distortion of myocardial fibers	–	–	+ +	+ +
Myocardial cytoplasmic degenerative changes	–	–	+ + +	+ +
Myocardial Nuclear degenerative changes	–	–	+ + +	+ +
Necrosis	–	–	+ +	+

(–) Normal, (+) mild injurious effect, (+ +) moderate, and (+ + +) severe

**Fig. 5:**
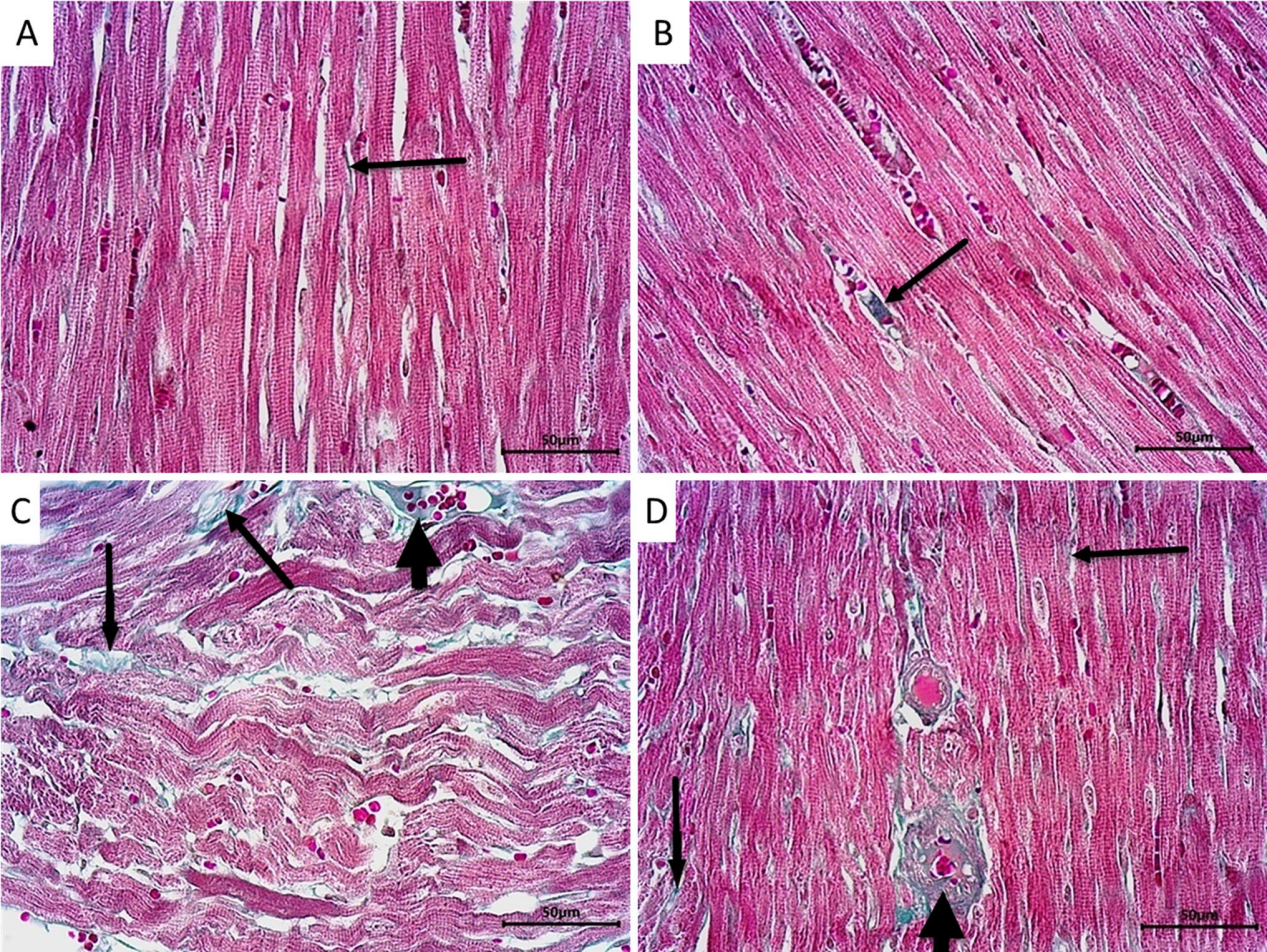
Photomicrographs of rat hearts stained with Masson’s Trichrome demonstrating collagen fibers deposition presented by green color. Sections from control (**A**) and LPZ-treated (**B**) rats show normal-appearing myocardial tissue with scanty collagen fibers in between the cardiac muscle fibers (long thin arrows). Section from CPA-treated rats (**C**) showing distorted myocardial tissue with obvious collagen deposition between cardiac muscle fibers (long thin arrows) and around blood vessels (short thick arrow). Section from rat hearts treated with CPA and LPZ (**D**) showing mild deposition of collagen fibers between cardiac muscle fibers (long thin arrows) and around blood vessels (short thick arrow). Scale bar = 50 µm

In order to evaluate the collagen fibers deposition in between the cardiac muscle fibers, we employed Masson Trichrome staining. Sections from control (Fig. 5A) and LPZ-treated (Fig. 5B) rats showed normal-appearing myocardial tissue with scanty collagen fibers between the cardiac muscle fibers. Administration of CPA (Fig. 5C) was found to distort the myocardial tissue with prominent collagen deposition between cardiac muscle fibers and around blood vessels. Interestingly, treating the rats with both CPA and LPZ (Fig. 5D) showed mild deposition of collagen fibers between cardiac muscle fibers and around blood vessels.

**Fig. 6:**
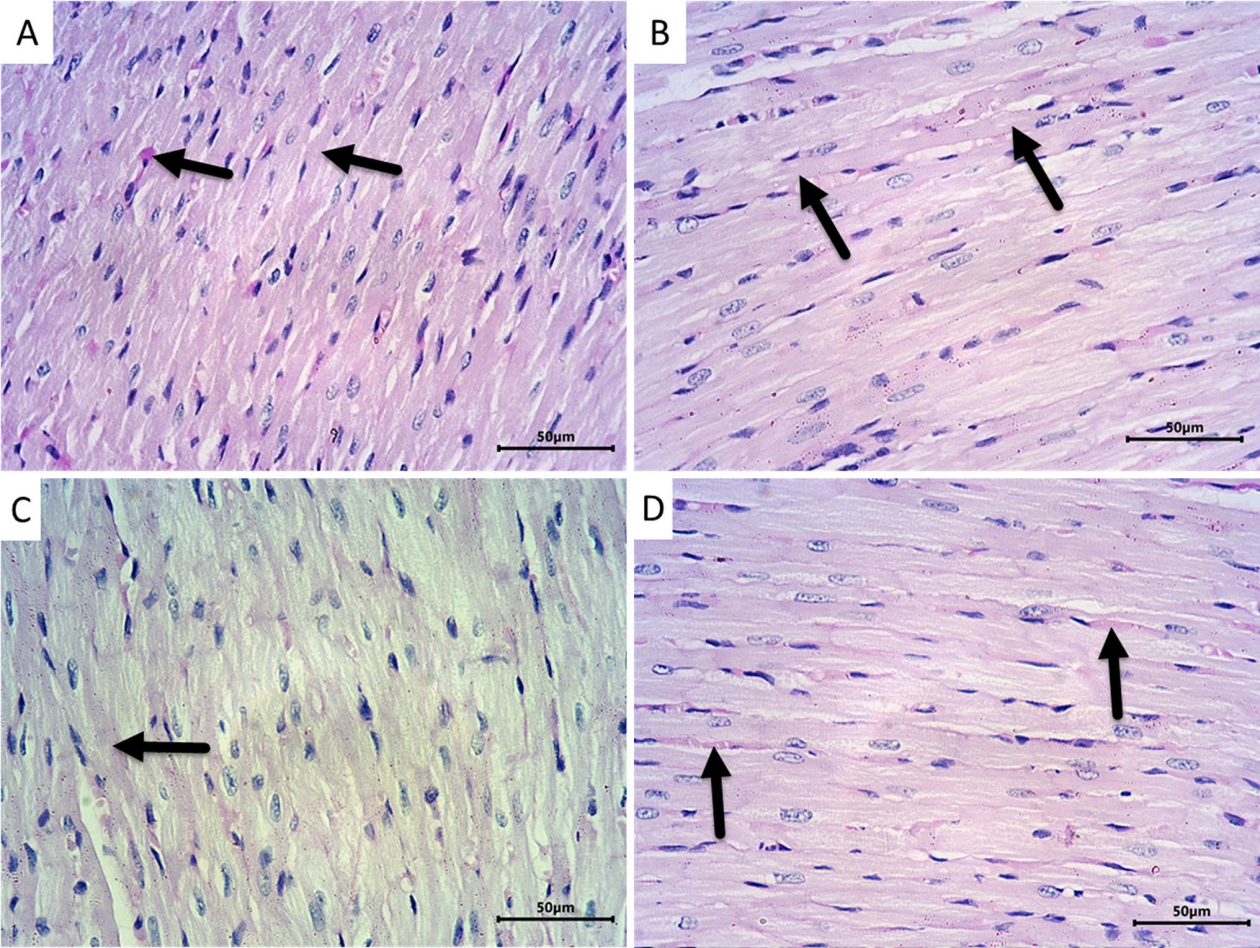
Photomicrographs of heart sections stained with PAS to evaluate glycogen amounts in cardiac muscle fibers. Sections from control (**A**) and LPZ-treated (**B**) animals show strong PAS reactions in the cytoplasm of cardiac muscle fibers (arrows). Treating rats with CPA **C** shows depletion of PAS reaction for glycogen in the cardiac muscle fibers cytoplasm (arrows). On the other hand, treating rats with LPZ after CPA toxicity **D** restored cardiac muscle fibers’ cytoplasm reaction for PAS near the normal (arrows). Scale bar = 50 µm

PAS histochemical reaction was employed to evaluate the glycogen deposition in the cytoplasm of cardiac muscle fibers. Sections from control (Fig. 6A) and LPZ-treated (Fig. 6B) animals showed strong PAS reactions in the cytoplasm of cardiac muscle fibers, indicating high glycogen deposition in cardiac muscle fibers. Treating rats with CPA (Fig. 6C) showed depletion of glycogen deposition in the cardiac muscle fibers cytoplasm. On the other hand, treating rats with LPZ after CPA toxicity (Fig. 6D) restored the glycogen amounts in cardiac muscle fibers’ cytoplasm near the normal.

### Effect of LPZ on pulmonary and cardiac oxidative injury induced by CPA

#### Effect of LPZ on lipid peroxidation and antioxidants

**Fig. 7:**
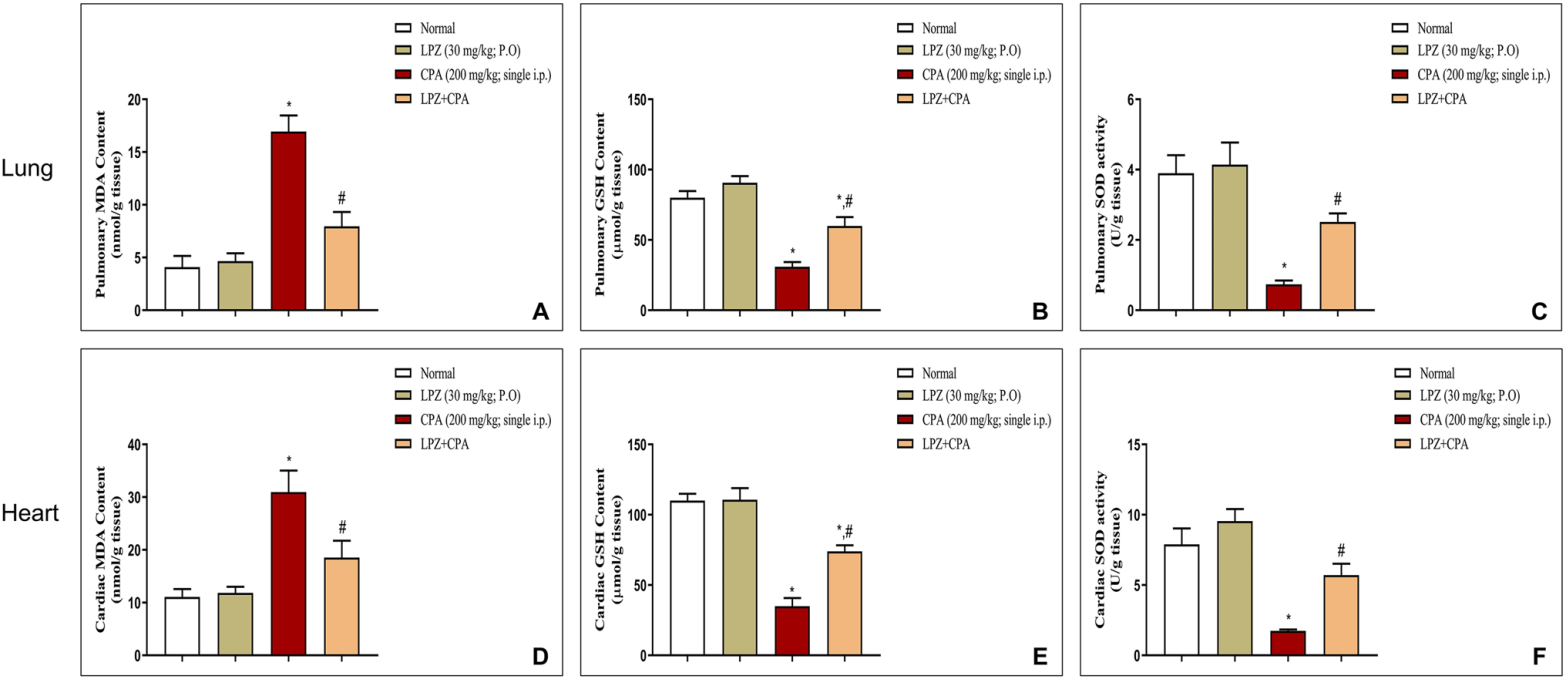
Effect of oral LPZ on the pulmonary and cardiac cellular oxidant/antioxidant balance after CPA challenge. Pulmonary (**A–C**) and cardiac (**D–F**) MDA, GSH, and SOD were estimated spectrophotometrically in 8 independent variables. Data are expressed as mean ± SEM. One-way ANOVA was performed for statistical analysis. ^*^P < 0.05 versus the normal control group. ^#^*P* < 0.05 versus the CPA control group

As shown in Fig. 7, the pulmonary and cardiac contents of MDA were significantly increased. In contrast, the pulmonary and cardiac contents of GSH, as well as the SOD enzymatic activity, were significantly reduced in rats given CPA alone. In comparison to CPA rats, LPZ treatment provided excellent protection against CPA-induced pulmonary and cardiac oxidative stress injury, as evidenced by a significant decrease in MDA content and boosting the antioxidants GSH and SOD in the lung and heart of the CPA-intoxicated rats.

#### Effect of LPZ on redox-sensitive pathways: PPARγ, Nrf2, HO-1, and cytoglobin in the heart and lung tissues

**Fig. 8:**
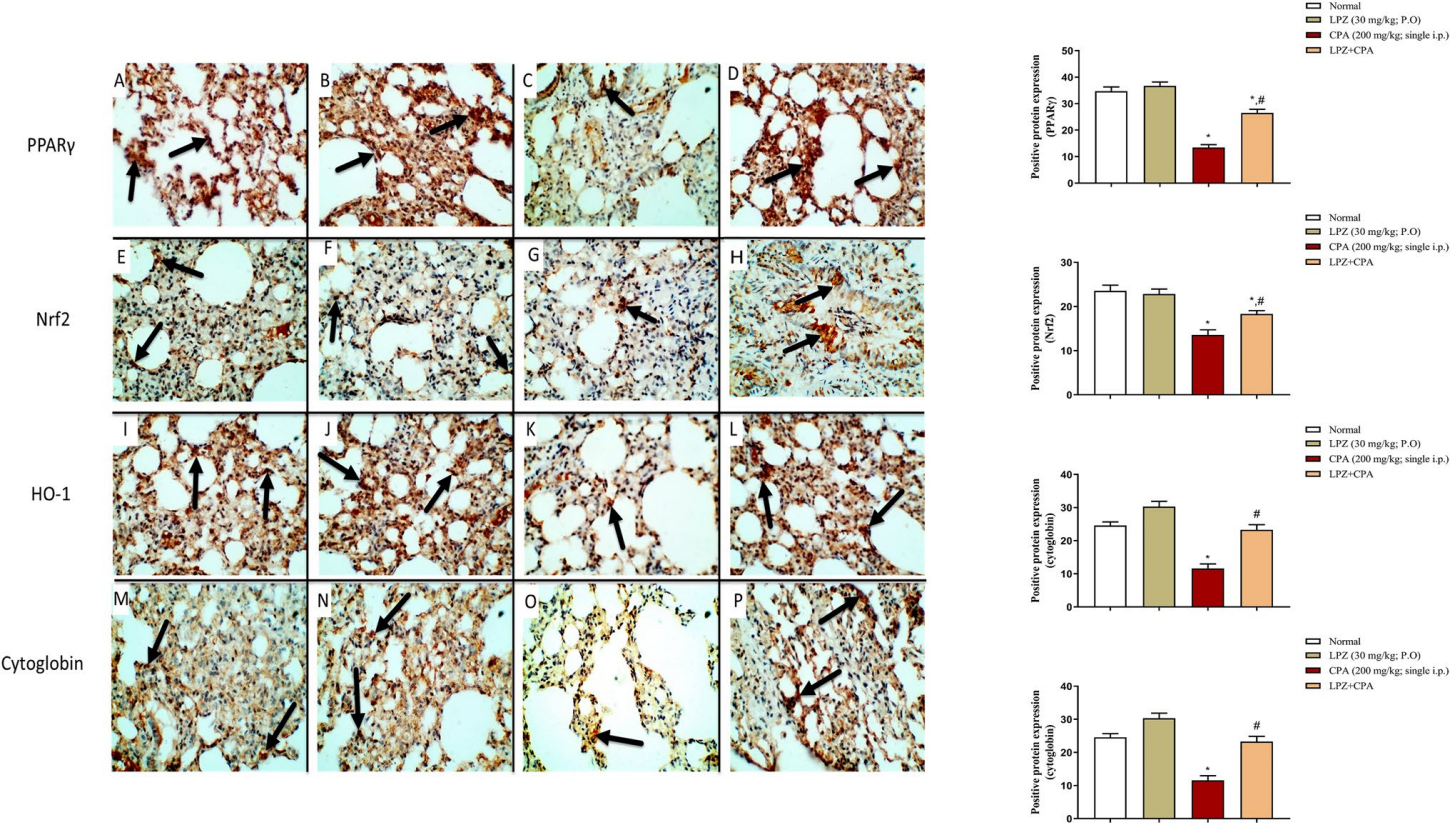
IHC expression of PPARγ (**A–D**), Nrf2 (E–H), HO-1 (**I–L**), and Cytoglobin (**M–P**) in rat lung tissues presented by brown color (arrows). In **PPARγ** groups, expression of PPARγ in lung tissue of control (**A**) and LPZ rats (**B**) is observed. In CPA-treated rats (**C**), arrows refer to weak PPARγ immunostaining. While in CPA + LPZ-treated rats (**D**), arrows show strong PPAR γ immunostaining similar to sections from control rats. In **Nrf2** groups, expression of Nrf2 in sections from control (**E**) and LPZ (**F**) rats is observed. In rats treated with CPA only (**G**), arrows show weak Nrf2 immunostaining in lung tissue. On the other hand, treatment with CPA and LPZ (**H**) shows intense Nrf2 immunostaining. In **HO-1** groups, strong immunostaining for HO-1 in control (**I**) and LPZ (**J**) groups is apparent. Weak HO-1 immunostaining is observed in CPA rats (**K**). While treating the rats with CPA and LPZ together (**L**) led to restoring the intense HO-1 expression in lung tissue compared to CPA alone. In **cytoglobin** groups, moderate immunostaining for cytoglobin in both control (**M**) and LPZ (**N**) rats was observed. Weak Cytoglobin immunostaining is expressed in lung tissue from CPA rats (**O**). While treating the rats with CPA and LPZ together (**P**) shows intense Cytoglobin immunoexpression in lung tissue compared to CPA alone. Data are expressed as mean ± SEM. One-way ANOVA was performed for statistical analysis. ^*^P < 0.05 versus the normal control group. ^#^*P* < 0.05 versus the CPA control group

**Fig. 9:**
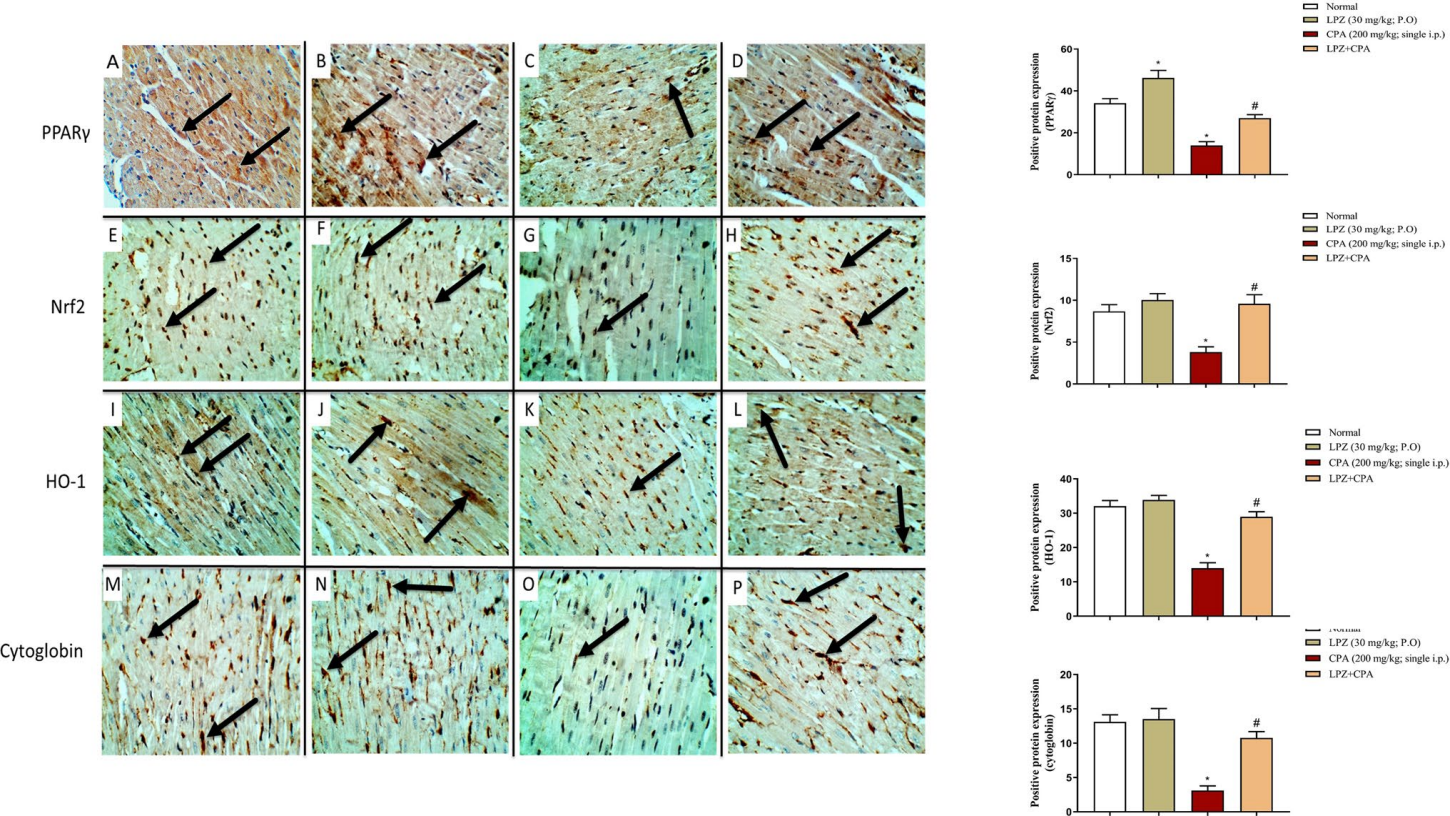
IHC expression of PPARγ (**A–D**), Nrf2 (**E–H**), HO-1 (**I–L**), and Cytoglobin (**M–P**) in rat heart tissues presented by brown color (arrows). In PPARγ groups, expression of PPAR γ in heart tissue of control (**A**) and LPZ rats (**B**) is observed. In CPA-treated rats (**C**), arrows refer to weak PPARγ immunostaining. While in CPA + LPZ-treated rats (**D**), arrows depict strong PPARγ immunostaining as in control rats. In Nrf2 groups, expression of Nrf2 in sections from control (**E**) and LPZ (**F**) rats. In rats treated with CPA only (**G**), arrows show weak Nrf2 immunoexpression in heart tissue. In contrast, treating the CPA rats with LPZ (**H**) showed intense Nrf2 immunostaining. In **HO-1** groups, the intense immunoexpression of HO-1 in control (**I**) and LPZ (**J**) groups is observed. Weak HO-1 immunostaining is shown in CPA rats (**K**). While treating the rats with CPA and LPZ together (**L**) leads to restoring the deep HO-1 expression in heart tissue compared to CPA alone. In **cytoglobin** groups, intense immunostaining for cytoglobin in both control (**M**) and LPZ (**N**) rats is referred to by arrows. Very weak Cytoglobin immunoexpression is shown in heart tissues from CPA rats (**O**). While, treating the rats with CPA and LPZ together (**P**) shows deep immunoexpression of cytoglobin in heart tissue compared to CPA alone. Data are expressed as mean ± SEM. One-way ANOVA was performed for statistical analysis. ^*^P < 0.05 versus the normal control group. ^#^*P* < 0.05 versus the CPA control group

The immunohistochemical technique is employed to demonstrate the effect of LPZ on CPA-induced alterations in the lung (Fig. 8) and heart (Fig. 9) expression of PPARγ, Nrf2, HO-1, and cytoglobin. The expression of PPARγ, Nrf2, HO-1, and cytoglobin was intense in the heart and lung tissues of control and LPZ-treated rats. In CPA-treated rats, PPARγ protein immunostaining was depleted, while treating the rats with CPA plus LPZ restored the intensity of the protein expressions of PPARγ, Nrf2, HO-1, and cytoglobin in the lung and heart tissues.

### Effect of LPZ on pulmonary and cardiac inflammation induced by CPA

**Fig. 10:**
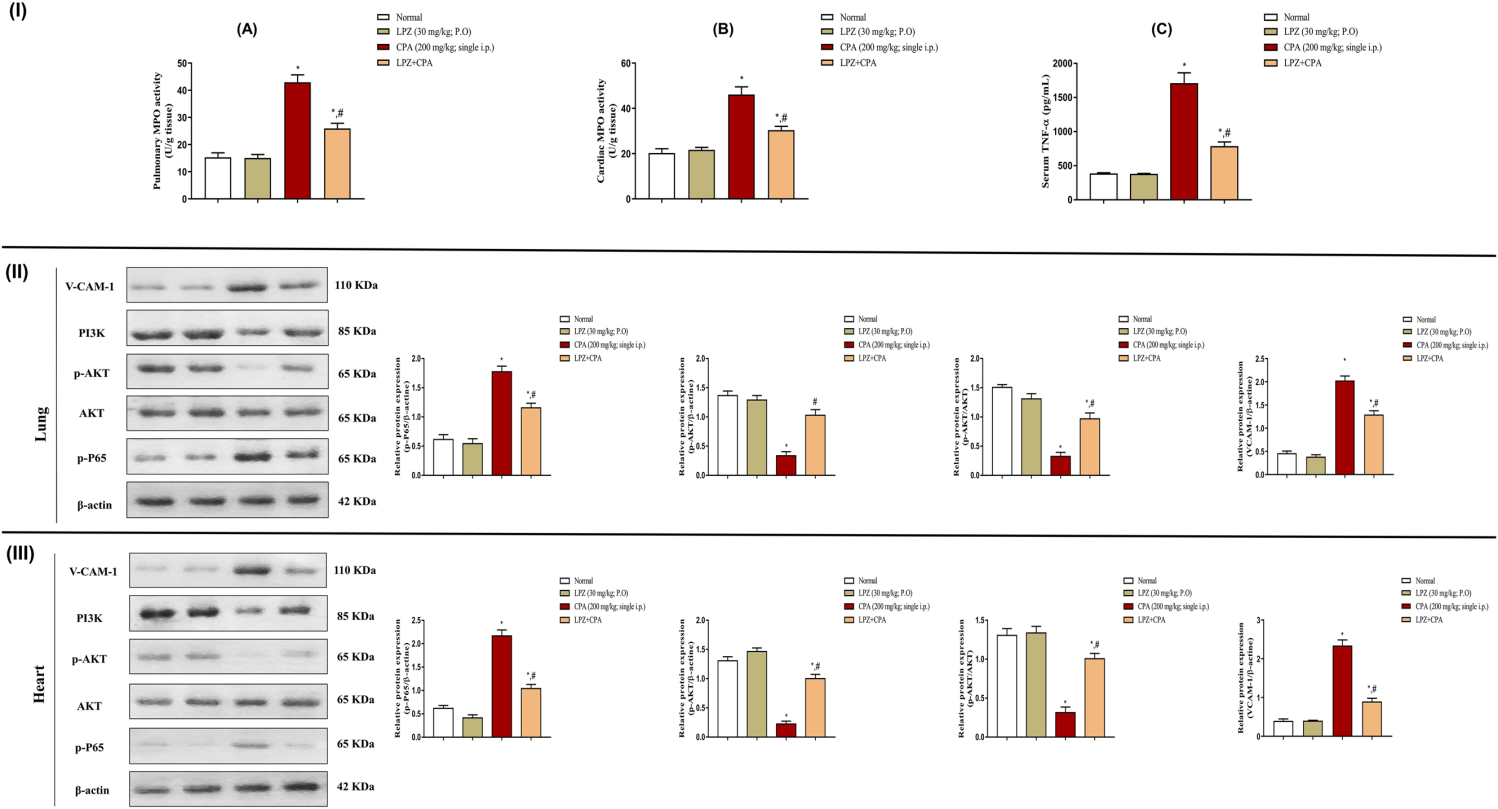
Effect of oral LPZ on the pulmonary and cardiac inflammatory burden and AKT/PI3K signal after CPA challenge. (**I**) Representative figures of pulmonary (**A**) and cardiac (**B**) MPO activity as well as serum TNF-α (**C**) level. (II) Representative figures of pulmonary western blots of p-P65, AKT, p-AKT, PI3K, and VCAM-1 and their semiquantitative analysis representation. (III) Representative figures of cardiac western blots of p-P65, AKT, p-AKT, PI3K, and VCAM-1 and their semiquantitative analysis representation. Data are expressed as mean ± SEM. One-way ANOVA was performed for statistical analysis. ^*^P < 0.05 versus the normal control group. ^#^*P* < 0.05 versus the CPA control group

As shown in Fig. 10, the inflammatory response elicited by CPA injection was investigated by measuring pulmonary and cardiac TNF-α levels as well as MPO enzymatic activity. TNF-α and MPO levels were significantly elevated in rats given only CPA. In contrast, treatment with LPZ effectively reversed these changes. Furthermore, the NF-κB signal was investigated to explore the underlined mechanism of CPA’s pulmonary and cardiac inflammatory perturbation. NF-κB was significantly elevated in the lung and heart of rats given CPA only compared to normal rats. Alternatively, these elevations were reduced considerably by LPZ administration with respect to CPA-intoxicated rats.

### Effect of LPZ on PI3K/AKT signal in the lung and heart of CPA-intoxicated rats

In this study, the expression of AKT, p-AKT, and PI3K in the lungs and hearts of rats given CPA was significantly higher than that of the normal rats. Co-administration of CPA and LPZ, on the other hand, increased AKT, p-AKT, and PI3K expression compared to CPA-treated rats (Fig. 10).

### Effect of LPZ on pulmonary and cardiac endothelial dysfunction induced by CPA

Cardiac and pulmonary VCAM-1 expression was investigated as a general marker for endothelial dysfunction. In parallel with our results, CPA injection caused a significant elevation in VCAM-1 expression as compared to normal control rats. However, pretreatment with LPZ significantly declined VCAM-1 expression in both hearts and lungs of rats as compared to CPA control rats (Fig. 10).

## Discussion

Cyclophosphamide is a commonly used chemotherapeutic agent. However, it is frequently associated with significant pulmonary and cardiac intoxications [5, 6]. LPZ is a proton pump inhibitor that has antioxidant and anti-inflammatory properties, according to recent studies [26, 41]. Despite the promising beneficial effects of LPZ, its impact on CPA-induced cardiac and lung toxicities has never been studied. This study investigated the protective role of LPZ on cardiopulmonary toxicity provoked by CPA and explored the underlined molecular mechanisms.

In the present study, the single CPA injection at a 200 mg/kg dose resulted in significant pulmonary and cardiac intoxication. In the lung, CPA causes pulmonary injury manifested by congestion, edema, hemorrhage with inflammatory infiltrate, narrowed alveoli with thickened septa, and swelling of type II pneumocytes. The same findings were observed in previous studies [5, 42, 43]. The edema and congestion in the epithelial cells are due to increased permeability of the alveolar capillaries caused by CPA [44, 45]. Also, our results revealed a significant increase in collagen deposition in the lung interstitium in the CPA group. This result can be explained by the ability of CPA to stimulate the alveolar macrophages to produce profibrotic cytokines [46]. Such cytokines lead to the proliferation and activation of fibroblasts with subsequently increased collagen deposition and alveolar collapse [47]. In parallel, the serum SGOT, ALP, LDH, and CK-MB levels were observed regarding cardiac injury by CPA. Histologically, CPA caused congestion, edema, hemorrhage, inflammatory infiltrate, fragmented myofibrils, vacuolation, pyknosis of nuclei, and myocyte disintegration in our study. Similar findings have been reported in previous studies [48–50]. Also, CPA caused prominent collagen deposition in between the cardiac muscle fibers. This finding was attributed to systemic and locally produced neurohumoral factors as a primary fibroblast growth factor that activates fibroblast proliferation and collagen synthesis [51]. On the other side, oral administration of LPZ potently lowered the SGOT, ALP, LDH, and CK-MB levels compared to untreated rats. Also, the histopathological findings induced by CPA were antagonized by the administration of LPZ, suggesting the protective action of LPZ on the heart and lungs. The protective effects of LPZ are widely contributed to reducing oxidative stress [29].

Previously, the administration of CPA induced significant cellular alterations and increased ROS production and lipid peroxidation [52]. ROS and lipid peroxidation can damage cellular components, including lipids, proteins, and DNA strands [53]. Also, ROS produced by CPA leads to compromised endothelial function [4], decreased ATP production [54], and increased mitochondrial dysfunction leading to cardiomyopathy [54, 55]. Regarding lung intoxication by CPA, acrolein disturbs the defense systems of the tissues and generates ROS, which damages the pulmonary cells [56]. During oxidative stress, enhanced production of ROS with pro-inflammatory cytokines can lead to cellular injury in the lung following CPA intoxication [57]. Also, free radicals scavenging enzymes, including SOD and GSH, are decreased significantly, leading to lung intoxication by CPA [30, 58]. Herein, in rats given CPA alone, the pulmonary and cardiac lipid peroxidation increased significantly, while the pulmonary and cardiac antioxidants markedly decreased. These actions are mediated by the downregulation of antioxidants PPARγ, Nrf2, HO-1, and cytoglobin signals. In contrast, in comparison to CPA-treated rats, LPZ treatment provided significant antioxidant protection against CPA-induced pulmonary and cardiac oxidative stress injury, as evidenced by a significant decrease in cardiac and pulmonary MDA contents and an increase in the antioxidants GSH and SOD in the CPA-intoxicated rats’ lungs and hearts. The protective effects of LPZ against oxidative stress are mediated via the Nrf2 pathway and were observed in previous studies [27, 59]. Nrf2 upregulation by LPZ results in upregulation in numerous antioxidant genes [14, 15, 60].

Another point of view is the impact of inflammation in pulmonary and cardiac intoxication by CPA. In pulmonary intoxication by CPA, activation of macrophages and neutrophils with the production of ROS and release of lysosomal enzymes caused by CPA cause disturbances of the normal cellular components [61] and thus injure the pulmonary endothelial cells and disturb the alveolar-capillary membrane causing edema [57]. In accordance, CPA induces hyperplasia of type II pneumocytes and contributes to the secretion of some cytokines. These cytokines act as chemoattractants leading to cellular infiltration [62], and this is similar to the present study, where dense inflammatory infiltrates were also observed. CPA also triggers the induction of cytokines that lead to cellular infiltration [7] observed in the present study. These structural changes in the cardiomyocytes were followed by functional distortion and depletion of glycogen storage in the cytoplasm of these cells. Biochemically, TNF-α and MPO levels were significantly elevated in rats given only CPA. These data parallel several previous studies [24, 63, 64]. In contrast, LPZ administration potently attenuated these changes mediated by down-regulating NF-κB compared to CPA-intoxicated rats.

Furthermore, the impact of CPA on PI3K/AKT signaling was assessed. Previously, a study done by Hassanein, Abd El-Ghafar, Ahmed, Sayed, Gad-Elrab, Ajarem, Allam, and Mahmoud [30] concluded that CPA induced heart injury by down-regulation of PI3K/AKT signal. Also, a recent study done by Abd El-Ghafar, Hassanein, Sayed, Rashwan, Shalkami, and Mahmoud [31] confirmed that CPA induced lung intoxication via downregulation of PI3K/AKT signal. These studies are consistent with our findings, which showed significant down-regulation in the PI3K, AKT, p-AKT, and the p-AKT/AKT ratio after injection with CPA. Treatment LPZ, on the other hand, potently attenuated CPA-induced cardiopulmonary oxidative injury while significantly up-regulating PI3K and p-AKT expression levels, as well as the ratio of p-AKT/AKT, without affecting total AKT levels.

Finally, we emphasized the antioxidant and anti-inflammatory effects of LPZ on cardiopulmonary injury induced by CPA by investigating its capability to prevent predicted endothelial dysfunction. It has been reported that oxidative stress and inflammation provoked endothelial dysfunction by interpreting membrane integrity [65]. Interestingly, VCAM-1 has been reported to have an important role in the development of endothelial dysfunction [66]. In the present study, LPZ can reduce cardiac and pulmonary VCAM-1 expression which in turn may reduce endothelial dysfunction.

## Conclusion

Lansoprazole could protect against CPA-induced cardiac and pulmonary intoxication by attenuating oxidative stress and inflammatory response. The protective action of LPZ could mediate by up-regulating PPARγ, Nrf2, HO-1, cytoglobin, and PI3K/AKT pathways concurrently with down-regulating inflammatory strategic controller NF-κB. These findings may provide a theoretical foundation for understanding LPZ’s role in CPA-induced cardiopulmonary injury. Finally, our work has raised concerns about a new scenario for the therapeutic effectiveness of giving LPZ to cancer patients receiving CPA in future.

## Data Availability

All data generated or analyzed during this study are included in this published article.
